# Quantitative genetic-by-soil microbiome interactions in a perennial grass affect functional traits

**DOI:** 10.1098/rspb.2022.1350

**Published:** 2023-01-25

**Authors:** Albina Khasanova, Joseph Edwards, Jason Bonnette, Esther Singer, Taslima Haque, Thomas E. Juenger

**Affiliations:** ^1^ Department of Integrative Biology, The University of Texas at Austin, 2415 Speedway #C0930, Austin, TX 78712, USA; ^2^ Department of Energy Joint Genome Institute, 1 Cyclotron Road, Berkeley, CA 94720, USA; ^3^ Lawrence Berkeley National Laboratory, 717 Potter Street, Berkeley, CA 94710, USA

**Keywords:** local adaptation, microbiome, *Panicum hallii*, plant functional traits, quantitative genetics, QTL

## Abstract

Plants interact with diverse microbiomes that can impact plant growth and performance. Recent studies highlight the potential beneficial aspects of plant microbiomes, including the possibility that microbes facilitate the process of local adaptation in their host plants. Microbially mediated local adaptation in plants occurs when local host genotypes have higher fitness than foreign genotypes because of their affiliation with locally beneficial microbes. Here, plant adaptation results from genetic interactions of the host with locally beneficial microbes (e.g. host genotype-by-microbiome interactions). We used a recombinant inbred line (RIL) mapping population derived from upland and lowland ecotypes of the diploid C4 perennial bunch grass *Panicum hallii* to explore quantitative genetic responses to soil microbiomes focusing on functional root and shoot traits involved in ecotypic divergence. We show that the growth and development of ecotypes and their trait divergence depends on soil microbiomes. Moreover, we find that the genetic architecture is modified by soil microbiomes, revealing important plant genotype-by-microbiome interactions for quantitative traits. We detected a number of quantitative trait loci (QTL) that interact with the soil microbiome. Our results highlight the importance of microbial interactions in ecotypic divergence and trait genetic architecture in C4 perennial grasses.

## 1. Introduction

Plants have evolved alongside microbes for millions of years and have formed intricate relationships with soil microbial communities via their root systems. Soil microbial community composition is shaped by soil abiotic conditions and varying soil types contain microbiomes with distinct taxonomic distributions [1,2]. To some degree, the root microbiome can be thought of as an extended phenotype of the plant. Plant–soil–microbiome relationships can influence plant traits and there is strong evidence that microbes can yield positive effects on plant performance directly or indirectly by impacting plant functional traits [3–5] or negatively as pathogens. Plant root-associated microbiomes impact root traits, can increase nutrient acquisition, provide indirect impacts on shoot traits (such as increasing shoot biomass) and promote tolerance to abiotic and biotic stress [6–9]. Growing plants of the same genotype in the presence or absence of differing sets of selected microbiomes produces a wide range of plant trait modulation [10,11]. Given the mounting evidence of microbial effects on plant growth and development, it's possible that plant microbial interactions also play a role in the process of local adaptation, by conveying benefits through specific affiliations with locally beneficial microbes that are not present in the ranges of other ecotypes [12].

Local adaptation is the outcome of natural selection driving adaptive phenotypic divergence across environmental gradients that results in specialization to habitats across a species range. Adaptive divergence in traits allows plant populations to persist and thrive across heterogeneous environments [13], but at a cost or trade-off [14] where native populations have higher fitness than foreign populations in each habitat. Ultimately, strong divergent selection and limited gene flow can result in the formation of disparate ecotypes [15], and as a result many plant species are composed of extremely varied ecotypes across their range. At a genetic level, local adaptation is the evolution of alleles that have varied fitness impacts across environmental heterogeneity, a type of genetic architecture termed genotype-by-environment interaction (G × E) [16]. Many studies focus on local adaptation and G × E in response to changing habitats or conditions [17–19], however, the relative contribution of abiotic and biotic factors in driving G × E is often unclear [20]. In the most detailed systems, genetic mapping has been used to identify genomic regions, or in a few cases the genes, underlying local adaptation and to explore the environmental factors driving G × E [21–23]. This approach has become widely used to study plant responses to abiotic stress and to understand plant trait plasticity [24,25], and a number of quantitative trait loci (QTL) exhibiting G × E have been discovered in natural and crop systems. Our previous work has shown substantial genetic variation between ecotypes in root system architecture and root/shoot relationships in a C4 perennial grass [26]. By analysing these known divergent traits between ecotypes in the presence of ‘home’ and ‘away’ microbiomes, we test which of these traits are predominantly under host genetic control, which are influenced by interactions with microbes, and whether there are important interactions between host genetics and the microbiome. Despite the robustness of this type of genetic analysis, the complexities of native microbial communities present several challenges to effectively studying these systems.

Microbial communities can be studied by reductionist approaches that ‘disassemble’ microbial communities or by holistic experiments that involve evaluating the interactions of plants with entire native and non-native microbial soil communities. Both reductionist and holistic approaches to evaluating plant–microbial interactions have their own sets of advantages and difficulties in both laboratory and field settings. Microbial communities are highly diverse and dynamic and many of their constituent strains are difficult to isolate and culture independently. Synthetic community (syncom) approaches involve small groups of isolated microbes for use in research, but such communities are incomplete representations of real-world environments. Additionally, these plant–syncom systems are often grown in synthetic conditions (e.g. on agar plates or in Magenta Boxes), that while useful in many contexts, are also incomplete representations. Conversely, manipulating microbial communities in natural conditions is nearly impossible due to a plethora of uncontrollable factors. A hybrid approach of introducing microbial communities to laboratory-grown plants through controlled and quantified inoculum derived directly from natural sources can bridge these two solutions [5]. While not without limitations, such studies allow an evaluation of the impact of the microbiome on plant traits that vary quantitatively in response to the presence of microbes in more controlled environments and help introduce tools like high-throughput phenotyping, genetic mapping, and genomic analyses to plant–microbiome studies [27].

To evaluate plant–microbiome interactions in this study, we used *Panicum hallii*, a diploid, C4, self-fertilizing, North American native perennial bunch grass that occurs across a large geographical range with diverse habitats and climate. There are two naturally occurring ecotypes of *P. hallii* that are classified as separate varieties: an upland xeric ecotype, *P. hallii* var. *hallii* (hereafter referred to as *hallii*) and a lowland mesic ecotype, *P. hallii* var. *filipes* (hereafter referred to as *filipes*). These ecotypes display trait divergence in a similar direction and magnitude to other perennial grass species with upland and lowland ecotypes, a pattern that is thought to be driven by adaptive evolution along precipitation gradients across a species range [26,28,29]. Xeric ecotypes typically have smaller root and shoot mass, shorter time to flowering, and higher specific leaf area and root mass ratio [26]. This suggests that xeric ecotypes use a rapid acquisition strategy to take up nutrients and flower quickly to escape summer droughts [26,30], while mesic ecotypes employ slow acquisition strategies with long-lasting thick leaves, larger more persistent roots and abundant aboveground foliage to take advantage of long growing seasons and consistent available moisture [26]. Many observations have shown that both ecotypes of *P. hallii* display a large degree of plasticity in several shoot traits in response to changes in abiotic factors including light [31] and precipitation [32], yet these differences are minor in comparison to the differences inherent between the ecotypes. Compared to abiotic factors, little is known about the importance or relative contribution of biotic environmental variation, especially soil microbiota, in shaping plant shoot and root traits in the *P. hallii* system and plants in general. Evaluating ecotype performance in the presence of ‘home’ versus ‘away’ microbiomes has the potential to indicate if these interactions are part of the driving forces behind local adaptation.

Here, we conducted a quantitative genetic experiment on a recombinant inbred population (RIL) derived from a cross between upland and lowland ecotypes of *P. hallii.* We collected microbiomes from the native soils of the RIL parents and used them to create microbial inocula. We then examined the impact of host genetics and these native soil microbiomes on root and shoot traits. Specifically, we used replicates of the RIL parents to investigate if inherent genetic differences in response to microbiome treatments exist between these genotypes, and we used RIL progeny to identify plant genomic regions contributing to microbial-mediated traits. In order to overcome the limitations of synthetic community approaches and the complexity of natural soils, we took a hybrid approach of inoculating sterilized soils with naturally derived microbial communities in a greenhouse setting. Specifically, we sought to answer four questions: (1) do *P. hallii* RIL ecotypes exhibit differential responses to variable soil microbiomes? (2) Does the presence of ‘home’ versus ‘away’ microbiomes generate GxE for plants traits, and can we map interactions with the microbiome to specific regions of the host genome? (3) To what degree is microbial community location of origin responsible for these effects? (4) Are observed microbiome-driven QTL effects consistent with putative ecotypic divergence of shoot and root traits? Overall, our experiment demonstrates the impact of living soil microbiomes on the quantitative genetic architecture of both root and shoot traits in *P. hallii,* and highlights the potential importance of microbiomes in local adaptation.

## 2. Material and methods

### (a) Plant material

We used a population of recombinant inbred lines (RILs) derived from a cross between *P. hallii* var. *hallii* (HAL2 genotype) and *P. hallii* var. *filipes* (FIL2 genotype) to evaluate the genetic basis of plant–microbiome interactions. FIL2 and HAL2 are selected genotypes that exemplify the plant trait variation between mesic and xeric ecotypes of *P. hallii*. The original cross generated a single F_1_ hybrid, which was used to generate a F_2_ population that was subsequently bred by single seed descent to the F_7_ generation [23]. Additionally, seed stock of the genotypes originally used as parents of the RIL have been maintained by single seed descent in the laboratory, and are used as the source of parental replicates in this study. Parents have full genome assemblies that are publicly available (https://phytozome-next.jgi.doe.gov/) [32]. The RIL population linkage map was constructed by shallow whole-genome resequencing and is congruent with the order of the physical genome [33]. Seeds of 293 F_7_ RILs and parents were sterilized, germinated and transplanted into treatment pots in a greenhouse located at the University of Texas at Austin (16 h days at 500 µE m^−2^ s^−1^, 28°C; 8 h nights at 24°C; full protocol in electronic supplementary material, appendix S1).

### (b) Microbial inoculum collection and treatment soil preparation

RILs and parental replicates (FIL2 and HAL2) were grown in the presence and absence of native microbiome inoculations obtained from the locations where parent genotypes were originally collected (Lady Bird Johnson Wildflower Center (WFC) in Austin, TX for var. *hallii* (HAL2) and the Corpus Christi Botanical Garden (CCBG), in Corpus Christi, TX for var. *filipes* (FIL2)). The Austin soil is a reddish rocky clay/silt and the Corpus Christi soil is a grey sandy loam; specific nutritional and mineral contents of each soil are given in electronic supplementary material, table S1. Soils for inocula were collected during late spring of 2018 from areas where *P. hallii* was present by clearing the soil surface of plant matter, collecting soil by shovel to a depth of 25 cm, and subsequently removing any root material present in the sample. Root material was removed to limit the impact of other native grasses and forbs on our inoculant, as our intention was for the inoculate to reflect the local bulk soil microbiome rather than the root microbiome enriched by a diverse plant community.

To create soil microbial inoculation treatments, we mixed 1% by volume of native soil (to minimize the effect of nutritive and textural soil properties from the inoculum) with a twice-autoclaved horticultural soil mix of compost, decomposed granite and vermiculite (Thunder Dirt, Geo Growers, Austin, TX), and incubated it for two weeks in closed 400 l plastic containers (as in [34]) in a greenhouse located at the University of Texas at Austin (16 h days at 500 µE m^−2^ s^−1^, 28°C; 8 h nights at 24°C). We selected this particular commercial soil mix because it homogenizes well and facilitates easy root extraction and cleaning. For soils utilized in the control treatments, the 1% native soil was twice autoclaved over a 24 h period before mixing and subsequent two-week incubation. Given the nature of this large-scale greenhouse experiment under an open-air environment, true sterility of the control treatments is not possible, and thus we refer to the treatments by their native soil inoculum source: microbiome treatments as HAL2 inoculated (HI) and FIL2 inoculated (FI) (soils that are intended to reflect the native microbiomes), and control treatments as mock HAL2 inoculated (MHI) and mock FIL2 inoculated (MFI) (soils that are intended as controls). Nevertheless, we feel this system allows us to assess the holistic impact of soil microbes, above and beyond what could be obtained from studies of individual microbes or experiments conducted under more artificial conditions.

### (c) Microbial DNA extraction and 16S rRNA gene sequencing

To characterize the microbial community composition of native locations, we collected samples of root rhizosphere and root endosphere from eight haphazardly selected var. *hallii* (HAL2) individuals growing at Austin (WFC) and nine var. *filipes* (FIL2) individuals growing at Corpus Christi (CCBG) in spring of 2019; we note that in this natural sampling scheme, location and genotype are confounded. At the time of collection, plants were in the early panicle emergence stage and in at least their second season of growth. Additionally, five bulk soil samples (all plant material removed) from each site were collected in areas adjacent to living *P. hallii* plants. These samples were collected at the same time of year and from the same locations as the bulk soil used for inocula, although 1 year later. DNA was obtained with the DNeasy PowerSoil Pro Kit (Qiagen, Hilden, Germany) for all 44 collected samples. 16S ribosomal RNA gene regions were amplified using the 515F-806R primer pair, barcoded and sequenced on the Illumina Novaseq platform on the SP flowcell using 2 × 250 reads. To characterize microbial community composition of the treatments in the greenhouse experiment, this procedure was repeated on rhizosphere, root and soil samples taken at harvest (prior to panicle emergence after six weeks of growth) from seven replicates of each parent in each treatment (four treatments (HI, FI, MHI, MFI) × 14 parents (HAL2 and FIL2) × three compartments (rhizosphere, root, and soil) = 168 samples).

### (d) Experimental design

Each treatment contained 293 RILs and seven replicates of each parent for a total of 307 plants per treatment in the experiment (four treatments × 293 RILs + 56 parents = 1,228 plants). Incubated soil for microbial and mock treatments was transferred to 950 ml 3″ × 8″ Mini-Treepots (Stuewe and Sons, Tangent, OR), which were lined with sterile plastic bags perforated at the bottom to allow water drainage and facilitate easy root system removal. Pots for all treatments were randomized in a single block design and left in the greenhouse for acclimation in open air for two weeks before seedlings were transplanted. Pots were rotated on the greenhouse bench weekly to reduce effects of micro-environmental variation and plants were watered with UV sterilized tap water for the duration of the experiment. UV sterilized water was used to reduce the contamination of experimental pots with non-inocula sources of microbes. We acknowledge that foreign DNA may have been present in this water source; however, the same source of water was utilized for the duration of the experiment across all treatments.

### (e) Harvest and phenotyping

Plants were harvested over a 5-day period after six weeks of growth at the tillering stage prior to panicle emergence. Individual plants were extracted from pots by pulling the plastic bag to prevent root damage, and plants with attached roots were removed from the soil by shaking over wire mesh. Root rhizosphere samples for parents were collected by dipping root systems into sterilized 50 ml tubes filled with 1 × phosphate buffered saline. Plants were then hung by the shoot base on a clamping apparatus, and soil particles were removed from the root system with a spray of UV-sterilized water. Roots were then separated from shoots and preserved in 90% ethanol for up to three months for future phenotyping. Tiller number of each plant and flag leaf area of each main tiller was measured. Shoot and leaf tissue were dried at 55°C and weighed separately to obtain aboveground biomass, and to calculate specific leaf area (SLA; fresh leaf area/dry mass of the leaf (cm^2^ g^−1^)).

Root systems were phenotyped by first scanning the entire intact root system, and subsequently isolating and scanning one nodal root with attached lateral roots on an EPSON 12000XL flatbed scanner (Epson America, Inc., San Jose, CA, USA) calibrated for use with WinRhizo Pro 2019 root image analysis software (Regent Instruments Inc., Canada) (full description in electronic supplementary material, appendix S1). Nodal roots are post-embryonic roots that provide support and the majority of nutrients to tillers. For parents, a small portion of the central root system was frozen for DNA extraction and PCR amplification to determine root endosphere microbial community composition. The remaining roots were collected and dried for 96 h at 55°C, to obtain belowground biomass, and to calculate root mass ratio (RMR; root biomass/total biomass).

### (f) Microbial sequence analysis

Demultiplexed sequences were trimmed to remove adapter and primer binding sites using Cutadapt [35]. Amplicon sequence variants (ASVs) were inferred using DADA2 using the following parameters: forward and reverse reads were trimmed to 210 and 190 bp, respectively; maximum expected errors allowed in each read was set to 2 for both forward and reverse reads; read pairs were removed if one or both reads were less than 170 bp [36]. Errant ASVs due to chimerization were detected using the ‘consensus’ method in DADA2 and discarded. Any ASV with a sequence length of greater than 256 bp or less than 250 bp were discarded. Taxonomic classifications were assigned to each ASV using DADA2's assignTaxonomy () function using the Silva reference database (v. 132) [37].

Microbiome data were analysed in R software [38]. ASVs assigned to mitochondrial and chloroplast lineages were discarded prior to normalization. For principal coordinates analysis (PCoA) and phylum level abundance statistics, raw counts were normalized to account for differences in sequencing depth between samples by dividing each ASV count by sequencing depth of a particular sample and multiplying by 1000 to place the counts on a per mille scale. Principal coordinate analyses were conducted using the capscale () function in the package Vegan [39]. Bray Curtis dissimilarity on log2 transformed relative abundances was used for all PCoAs unless otherwise noted. Shannon index was calculated using diversity () function in Vegan. Differential abundance of aggregated phylum abundances was performed using linear models on log2 transformed relative abundances. Differential abundance of ASVs between conditions was conducted using DESeq2 on raw counts [40]. To compare the relative abundance differences in plants from the field, we coded a factor combining field site and root compartment (for example, ‘Austin_Rhizosphere, Corpus_Root, etc.’). We included this combined factor into DESeq2's design formula to model each ASVs relative abundance as a function of compartment and field site (ASV_X_ ∼ combined_factor). We then contrasted across conditions to identify ASVs with significantly different relative abundance. The same method was used in the greenhouse experiment to identify differentially abundance ASVs between conditions.

### (g) Data and QTL analysis

Plant trait data from all parental replicates was analysed to test genotypic and soil microbial inoculation treatment effects on plant morphological traits. We fit factorial linear models using PROC MIXED in SAS [41] consisting of genotype (G) treatment (T), and genotype × treatment (G × T) interactions as fixed effects. We analysed data from the RIL population using factorial linear mixed models with PROC MIXED in SAS. Here, RIL genotypes were considered a random effect. Preliminary analysis did not show any significant differences for all measured phenotypic traits (in all cases, *p* > 0.113) between MHI and MFI treatments for parents and RILs, thus the average between them was used for this and all subsequent analyses (hereafter referred to as the mock inoculated (MI) treatment). Pearson correlation was conducted by using ggpairs() function in the package GGally [42].

To explore the impact of the microbiome on the quantitative genetic architecture of our measured traits, we also fit linear mixed models testing for G × T using the sommer package [43] in R based on the additive and epistatic relationship matrix determined from the genotypic data of the RIL. Our approach competed a simple ‘base’ model including additive genetic variance (*V*_a_), additive*additive epistatic variance (*V*_aa_) and a fixed treatment effect to more complex models that allowed either the additive genetic variance (*V*_a_), additive*additive epistatic variance (*V*_aa_) or the residual to vary by the microbiome inoculation treatment (HI, FI, MI). Models were compared with AIC and LIK and assumed no covariance among treatments. We calculated broad-sense heritability (*H*^2^) as (*V*_a_ + *V*_aa_)/*V*_p_ and made variance components and model comparisons.

The observation of different QTL effects under different treatment conditions provides evidence for QTL × treatment interactions. There are a number of potential statistical strategies for detecting the occurrence of QTL × treatment interactions [16]. To detect QTL present in the HI, FI and MI treatments, we completed QTL mapping on RIL values (electronic supplementary material, table S2) in R using the R/qtl package [44] in each environment separately (script: https://github.com/AlbinaKh/QTL_P.hallii_Native_Microbiome). When quantitative trait data distributions were not normally distributed, data were log transformed (tiller number, root number, shoot biomass, root biomass, root diameter, root mass ratio, lateral root length). We used calc.genoprob with step = 2 and map.function = ‘kosambi’ to calculate genotype probabilities every two cM. The following functions were used to determine the position of QTL and to conduct the calculation of estimates for additive effects and effects of epistasis (additive-by-additive interaction between quantitative trait loci). Penalties for main effects for each trait were calculated with the scantwo function on 1000 permutations, and the stepwise QTL function was used to conduct a forward–backward search accounting for epistasis with a maximum of seven QTL that optimized the penalized LOD score criterion. For all traits the alpha was set at 0.05 as a threshold for type 1 error rates based on permutation to detect main QTL. We consider these genomewide scans within environments as a tool to discover genomic regions of interest using a relaxed threshold of alpha = 0.1. We used the qtlStats () function to calculate the 1.5 LOD drop interval of QTL [45].

We tested for QTL × treatment interactions by building multiple QTL models for each trait separately using the PROC MIXED procedure of SAS. We completed these additional analyses, as there are no existing tools which easily allow genomewide scans for epistatic-by-treatment interactions. First, in R, we used fill.geno with the method = ‘maxmarginal’ and min.prob = 0.95 to fill in missing genotypic data. Then, in SAS, these data were used in a full linear model including the main and interactive effects (marker, treatment, marker × treatment, marker × marker and marker × marker × treatment) of all QTL identified in the initial analysis in R (discovered with alpha = 0.1). The markers and their interaction with the microbial treatments were treated as fixed effects [46], and RIL was included as a random effect. Marker × treatment interaction indicates QTL × treatment interaction, marker × marker interaction represents epistasis averaged over the treatments, and marker × marker × treatment interaction indicates treatment-specific epistasis. To account for multiple testing, we used the Holm–Bonferroni method for multiple comparisons, and present both significant (with corrected alpha 0.05) and suggestive effects (with corrected alpha 0.1) for discussion. To test the significance of individual marker alleles at each treatment, we used the SLICE function in SAS as tests of simple effects (partitioned analysis of the LS-means for an interaction) [47] for all significant and suggestive marker × treatment interactions.

## 3. Results

### (a) Treatment drives bacterial community composition

**Figure 1.**
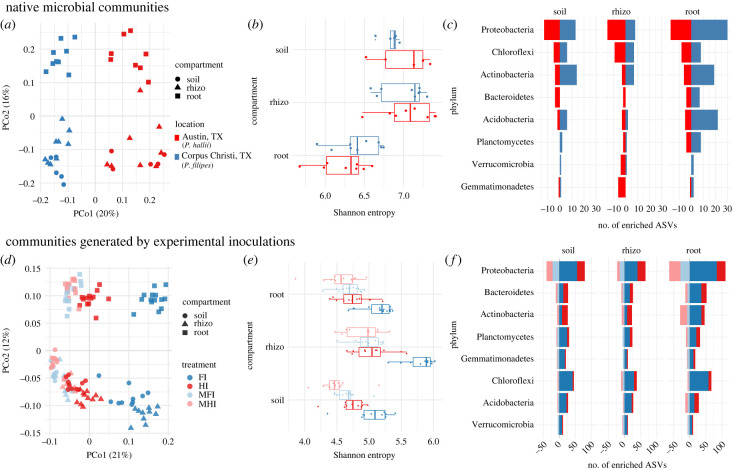
Parental genotypes grown under natural conditions host distinct microbiota. (*a*) Principal coordinate graph based on Bray–Curtis dissimilarities; (*b*) Shannon diversity of samples from native parental habitats; (*c*) number of ASVs with differential abundance between parental habitats broken down by phylum where bars to the left indicate number of ASVs enriched in the HAL2 native habitat, while bars to the right indicate number of ASVs enriched in the FIL2 native habitat. The colour scheme in (*b*) and (*c*) is consistent with panel (*a*). Microbial treatments differ significantly in community composition for plants growing in the greenhouse. (*d*) Principal coordinate graph based on Bray–Curtis dissimilarities; (*e*) Shannon diversity of greenhouse samples; (*f*) number of differentially abundant ASVs when comparing HI versus FI soil inoculum for both native and mock treatments, where bars to the left indicate the comparison for the mock treatments while bars to the right indicate native treatments. The colour scheme in (*e*) and (*f*) is consistent with panel (*d*).

We used 16S rRNA gene amplicon sequencing classified into ASVs to characterize both the native microbial communities and communities generated by experimental inoculations as a representation for overall biotic differences. For parental genotypes growing under natural habitats, PCoA of bacterial ASV counts revealed strong location/genotype and compartment effects (i.e. soil, rhizosphere, and root) across axes one and two, respectively (figure 1*a*). Permanova mirrored these results with location/genotype explaining the most variance (*R*^2^ = 0.18, *p* < 0.001) and compartment explaining the second most (*R*^2^ = 0.16, *p* < 0.001; table 1). Microbiota varied significantly in diversity between compartments, but not between location (as measured by Shannon Index, figure 1*b*). Phylum level distributions were overall consistent between microbiota of plants growing at the two natural locations with *Proteobacteria*, *Actinobacteria* and *Acidobacteria* being dominant members (electronic supplementary material, figure S1a), which is congruent with results from previous root-associated microbiome studies [48–51]. Only three relatively low abundance phyla displayed significant differences between location/genotype: WPS-2 and *Entotheonellaeota* in the rhizosphere, and *Rokubacteria* in the root (electronic supplementary material, figure S2). Conversely, microbiota from the two locations were more divergent at the ASV level: 304 unique ASVs were identified as being differentially abundant between location/genotype depending on compartment (78 in soil, 72 in rhizosphere and 154 in roots; figure 1*c*).

**Table 1.** PERMANOVA partitioning and analysis of 16S community composition of native populations of *P. hallii* genotypes and experimental plants grown in the greenhouse.

collected	effect	d.f.	SS	F.Model	*R*^2^	*p*-value
native environment	location/genotype	1	1.8131	11.3967	0.18114	0.001***
	compartment	2	1.6509	5.1887	0.16494	0.001***
	location/genotype × compartment	2	0.4998	1.5708	0.04993	0.033*
greenhouse	genotype	1	0.1465	1.6926	0.00572	0.058
	treatment	3	8.2427	31.7666	0.32195	0.001***
	compartment	2	4.8768	28.1748	0.19036	0.001***
	genotype × treatment	3	0.3252	0.2526	0.01269	0.118
	genotype × compartment	2	0.1412	0.8158	0.00551	0.731
	treatment × compartment	6	0.3404	3.5576	0.07211	0.001***
	genotype × treatment × compartment	6	0.3404	0.6554	0.01329	0.997

We next analysed microbiota acquired under experimental conditions in the greenhouse by sampling rhizosphere and roots from the RIL parents, along with soil from unplanted pots. PCoA revealed that microbiome inoculation treatment and compartment significantly impacted microbiota composition (figure 1*d*). Permanova mirrored these results with treatment explaining the most variance (*R*^2^ = 0.32, *p* < 0.001), and compartment explaining the second most (*R*^2^ = 0.19, *p* < 0.001; table 1). Microbial diversity was also impacted by compartment and microbiome inoculation treatment: in general, plants grown in treatments with native soil microbiome hosted microbiota with greater Shannon index compared to plants with mock-inoculated treatment, (figure 1*e*). Plant genetic variation did not alter the microbiomes (*R*^2^ = 0.008, *p* = 0.1). As expected, when comparing the effect of inoculum source within microbiome and mock treatments, we found that microbial communities of plants and soil grown in mock-inoculated treatments were significantly more similar than in the microbiome-inoculated treatments, and this effect was consistently independent of compartment (electronic supplementary material, figure S3). Similar trends were observed at the phylum level, where there were many more differentially abundant phyla by native soil source microbiome inoculation versus mock treatments (electronic supplementary material, figure S1b, c; 17 differentially abundant phyla in native versus two in mock treatments). When identifying ASVs whose abundance was impacted by soil inoculation treatment, many more ASVs were differentially abundant in comparisons between native soil inoculum treatment compared to mock (figure 1*f*). When analysed together, we found that greenhouse and field microbiomes formed distinct communities, yet were still identifiable by soil source (electronic supplementary material, figure S4a, more in appendix S1). These results indicate that heat sterilization of soil inoculum dampens the effect of soil source on compositions of the resulting microbiome, and that plants inoculated with native microbiota host significantly different communities in their rhizosphere and roots. We acknowledge that these are not re-creations of native microbial communities, likely because the horticultural media differs texturally and nutritionally from the native soils. We also recognize there are caveats to 16S RNA profiling [52,53]; nevertheless, 16S profiling shows that this method creates significantly different treatment communities which are influenced by the native inoculum source.

### (b) Effect of microbiome inoculation on parental traits

**Figure 2.**
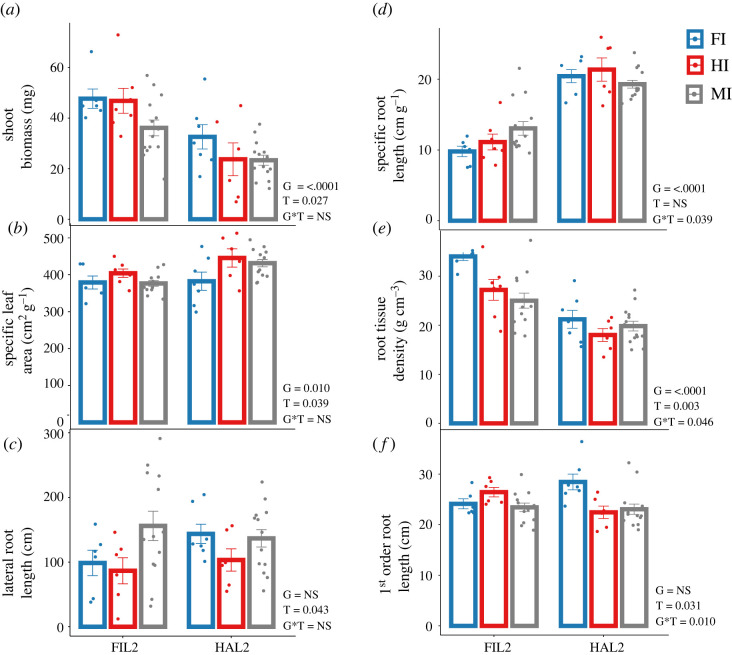
Effect of plant genotype (G), microbial treatment (T) and their interaction (G*T) on plant functional traits. Traits: (*a*) shoot biomass; (*b*) specific leaf area; (*c*) lateral root length; (*d*) specific root length; (*e*) root tissue density; (*f*) first order root length. Data are means ± s.e. and *p*-values are presented from the omnibus tests for each factor in the factorial model.

Trait differences among parents were driven by plant genotype, treatment and genotype × treatment interactions. Parents differed in shoot and root traits across all treatments. For example, FIL2 produced 1.64-fold more shoot biomass (*p* < 0.0001), 1.98-fold more root biomass (*p* < 0.0001), 1.80-fold lower specific root length (SRL) (*p* < 0.0001) and 1.46-fold higher root tissue density (RTD) (*p* < 0.0001) relative to HAL2 (figure 2*a,d,e*; electronic supplementary material, table S3; table S4). These results mirrored earlier descriptive studies of *P. hallii* genotypes [29,54], including studies of the same shoot and root traits studied in this current work [26]. Treatment also had a significant effect on plant traits (figure 2*a–c,e,f*; electronic supplementary material, table S3, table S4). For example, plants grown in soils that were inoculated with native soil microbiome had greater shoot biomass (1.35-fold more biomass in FI and 1.17-fold more in HI treatments relative to the MI treatment (*p* = 0.027)), lower lateral root length (1.2-fold less in FI and 1.53-fold less in HI relative to MI (*p* = 0.046)), and showed inocula source specific changes in specific leaf area (SLA) (1.05-fold increase in HI and 1.06-decrease in FI relative to MI (*p* = 0.039)). Importantly, we also identified several G × T interactions (figure 2*d–f*; electronic supplementary material, table S3, table S4). For example, SRL of FIL2 decreased 1.17-fold under HI and 1.33-fold under FI relative to MI inoculum source, while HAL2 showed 1.1-fold increase in SRL under HI and no change under FI relative to MI (*p* = 0.039; figure 2*d*; electronic supplementary material, table S3, table S4). Root tissue density (RTD) of FIL2 increased 1.1-fold under HI and 1.36-fold under FI relative to MI, while HAL2 showed 1.1-fold decrease under HI and 1.1-fold increase under FI relative to MI (*p* = 0.046; figure 2*e*; electronic supplementary material, table S3, table S4). In total, seven traits showed genotype differences between parents, five traits were affected by microbial treatment, and three traits had significant G × T interaction (figure 2; electronic supplementary material, table S3, table S4).

### (c) Effect of microbiome inoculation on quantitative genetic architecture of plant traits

Many plant traits showed strong positive correlations for phenotypic traits in the RIL population that were consistent between microbial treatments (electronic supplementary material, figure S5). The impact of the microbiome on the quantitative genetic architecture of our measured traits was evaluated by comparing ‘base’ and ‘G × T’ linear mixed models. In 11 out of 12 cases, the G × T models were favoured by AIC and log-likelihood ratio tests (electronic supplementary material, table S5). However, broad-sense heritability *H*^2^ was low for most traits (ranging from 0.01 to 0.18; electronic supplementary material, table S3). Overall, we document considerable evidence that the microbiome modifies the expression of quantitative genetic variation in *P. hallii*.

**Figure 3.**
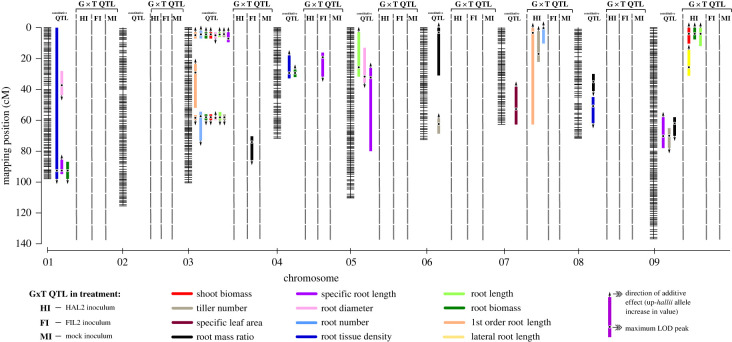
Genetic map of the *Panicum hallii* RIL population with locations of significant constitutive and G × T QTL for measured traits.

A total of 33 constitutive QTL were identified for 12 traits that were robust across environments (figure 3; electronic supplementary material, table S6, table S7). The additive effects of each QTL explained from 2.9–22% of trait variation (electronic supplementary material, table S6). Of these 33 QTL, six QTL occupied unique positions in the genome. The confidence intervals of all other QTL overlapped or colocalized with at least one other QTL. Seven traits (shoot biomass, tiller number, lateral root length, root diameter, root number, root biomass and total root length) had 14 QTL with overlapping confidence intervals grouped into two hotspots on chromosome three (figure 3; electronic supplementary material, table S6). The hotspot located on 3@4.3 (chromosome number @ centimorgan) showed an additive effect in the direction of parental genotype divergence, while the other hotspot located on 3@58 showed an additive effect opposite the direction of parental divergence. We also found significant epistatic interaction between these two hotspots. Individuals possessing the *hallii* allele for the QTL on 3@58, masked the effects of their interactive QTL on 3@4.3 (electronic supplementary material, table S6). For two traits, shoot biomass and lateral root length, this epistatic interaction was present in each treatment, but the effect was stronger in MI treatment.

**Figure 4.**
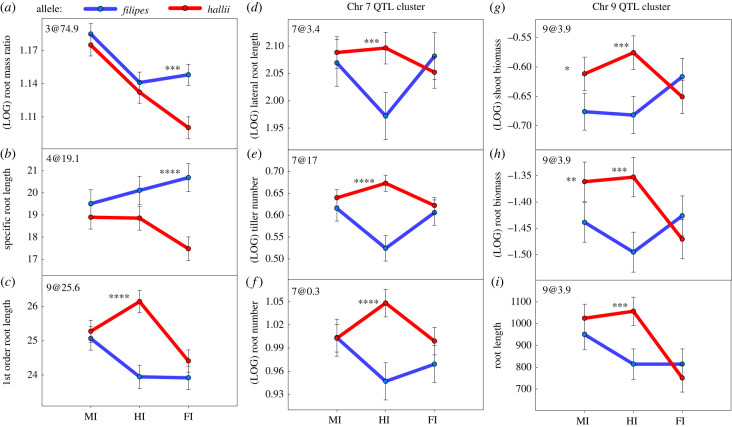
Tests for significant treatment × marker interactions for traits in multiple QTL models using PROC mixed in SAS. Panels: (*a*) root mass ratio at 3@74.9; (*b*) specific root length at 4@19.1; (*c*) first-order root length at 9@25.6; (*d*) lateral root length at 7@3.4; (*e*) tiller number at 7@17.0; (*f*) root number at 7@0.3; (*g*) shoot biomass at 9@3.9; (*h*) root biomass at 9@3.9; (*i*) total root length at 9@3.9. Significance for marker × treatment: **** < 0.0001, *** < 0.001, ** < 0.05, *0.05. Coloured lines connect LS-means for an allelic category in each treatment, the dots and error bars are LS-means ± 1 s.e. from the PROC mixed model using slice function in SAS.

Our experimental design allows us to detect inocula source-specific effects of our microbial treatments. A total of nine G × T QTL were identified related to the specific origin of the microbiome for nine traits (figure 3; electronic supplementary material, table S7, table S8). The additive effects of each QTL explained from 3.5–6.5% of trait variation (electronic supplementary material, table S8). Each of these QTL were analysed to directly test in which treatment they were present, and to estimate the direction and magnitude of their effects (figure 4*a–k*; electronic supplementary material, table S8, table S9). In the MI treatment, colocalized QTL on chromosome 9 for shoot biomass and root biomass were detected, with the *hallii* allele contributing to a higher trait value (figure 4*g,h*). In the FI treatment, QTL for RMR and SRL were detected, with the *filipes* allele contributing to a higher trait value (figure 4*a,b*). In the HI treatment, QTL for tiller number, root number, lateral root length, shoot biomass, root biomass, total root length and first order root length had allelic effects, with the *hallii* allele contributing to a higher value for all traits (figure 4*c–i*). Of these QTL, three have overlapping confidence intervals and are grouped into a hotspot on chromosome seven, and three are grouped together on chromosome nine (figure 4*d–i*; electronic supplementary material, table S8, table S9). Two of the three QTL present on chromosome nine were also detected in the MI treatment (figure 4*g,h*; electronic supplementary material, table S8, table S9).

## 4. Discussion

There is growing appreciation of the important and often complex interactions that exist between plants and their associated microbial communities. Exploring the genetic architecture of plant trait–microbiome interactions is an important step in determining if these interactions play a role in local adaptation and evolution. Here, we conducted a QTL study with a *P. hallii* RIL mapping population in soils inoculated with microbiomes from the original native habitats of the RIL parents and observed the impact of these microbiomes on plant traits and genetic architecture. We found that the soil microbiota in the native habitats of the two RIL parents are distinct, and that extant representatives of the original RIL parent populations obtained from these native habitats also possess distinct rhizosphere and root microbial communities. In this study, soils inoculated with native microbiomes altered both above and below ground traits in the RIL parents compared to controls. Some of these effects were dependent on the addition of either the HI or FI treatment; while for others, alterations occurred in both treatments. In the RIL population, we found 33 QTL for 12 measured traits that were robust and did not interact with the microbial treatment, suggesting that much of the trait variation was confined primarily to host genetics or unmanipulated environmental variation. However, we also found nine QTL that displayed G × T suggesting that some genetic variation in trait responses are due to plant–microbiome interactions. Overall, our study suggests that the genetic architecture of host functional traits is significantly impacted by microbial associations.

For the RIL parents, both above- and belowground traits were altered in the presence of microbiomes from native soil inocula (figure 2). For example, traits linked to resource acquisition such as specific leaf area (SLA) and specific root length (SRL) were altered in response to the presence of both microbial treatments. High SLA correlates with high nitrogen content and low structural investment in leaves, which yields high rates of photosynthesis and promotes rapid growth [55,56], a trait necessary in xeric environments with short seasons terminated by drought. This is analogous to high SRL, where plants produce longer and thinner roots with less structural input to search more rapidly and to greater distances for water [30]. SLA in both parent genotypes showed a response to inocula source specific native microbiomes: SLA was increased for plants with the HI microbiome and decreased for plants with the FI microbiome. This observed pattern in SLA is consistent with the directionality of ecotypic divergence in this system. Moreover, SRL showed G × T in response to microbiomes that was also concordant with the direction of parental trait divergence: with xeric adapted *hallii* showing higher SRL in the presence of both microbial treatments, while mesic adapted *filipes* showed lower SRL. We examined the RIL parents on the grounds that trait responses to microbiome treatments found in these genotypes should also be present in the RIL population and likely in the same direction. Our initial expectation was that many trait responses would be in the direction of genotype divergence correspondingly in their ‘home’ or ‘away’ treatments, and this was confirmed in some cases, but not in all. The directionality of responses in the parents and RIL population detected was quite diverse and complex, indicating that responses for each trait is independent to some degree.

For the RIL population we detected 33 constitutive QTL, and nine QTL interacting with native-derived microbiomes. Although many more QTL were detected without G × T, the effect sizes were similar across both constitutive and treatment interactive QTL groups. This indicates that although the majority of genetic variation that underlies genotype divergence in this population resides in the host, interactions with the microbiome make a significant contribution to ecotypic divergence. Two QTL for specific root length and root mass ratio showed inocula source specific G × T (figure 4*a,b*), where the *hallii* allele contributed to a lower phenotype compared to the *filipes* allele in the ‘away’ FI treatment but not in the ‘home’ HI treatment. These effects, present only in the mesic derived FI microbiome treatment, represent an alteration of root architecture and growth rate that is more common in mesic adapted plants.

Seven G × T QTL showed inocula source specific G × T to the HI treatment (figure 4*c–i*), where the *hallii* allele contributed to a high phenotype compared to the *filipes* allele in the ‘home’ HI treatment but not the ‘away’ FI treatment. Our previous study conducted with this RIL population at the panicle emergence stage suggested that xeric *hallii* employs a fast-acquisitive strategy for drought escape by acquiring nutrients rapidly and flowering quickly to enter dormancy before the onset of summer drought [26]. This is consistent with the current study conducted at the tillering stage, where plants with the *hallii* allele interacting with the HI microbiome produced more root and shoot biomass. This change in morphology is accomplished by the increased production of tillers with roots to support them. Additionally, root systems of RILs with these *hallii* allele QTL hotspots produced longer roots, putatively allowing increased foraging and resource acquisition. This complementary pattern of QTL interaction (*hallii* allele interacting with *hallii* microbiome) is in the directionality of trait divergence and is therefore consistent with microbial mediated local adaptation in this system. Most of these QTL are clustered in two ‘hotspots’ (where multiple QTL occur in the same genomic region). This common genetic control of genotype differentiating traits involving above and below-ground traits suggests that these factors interact with the HI microbiome in tandem, and that pleiotropic genes or linked genes with correlated effects may drive these genomic hotspots of correlated traits through interaction.

It is intriguing to speculate on the genes and molecular mechanisms underlying the host × microbiome QTL detected in our study. It could be that these QTL harbour genes that interact only indirectly with the host microbiome, perhaps through abundance of soil nutrients as modified by microbes. For example, certain soil microbes in our inoculates may alter the abundance or availability of soil nutrients with subsequent consequences for genetic variation in root or shoot growth. It may be that QTL are related to root exudates or root-released metabolites that may recruit or amplify key beneficial microbes with subsequent impacts on available nutrients. There are many examples of soil resource abundances of key nutrients impacting plant growth, including genes that demonstrate plastic responses to nutrient availability [57]. Alternatively, it may be that the genes within QTL intervals are involved in more direct interactions with microbes. For example, recent studies show that phytohormones, microRNAs and secreted peptides are known to recruit and foster the establishment of symbiotic arbuscular mycorrhizal fungi [58]. Moreover, Finkel *et al.* [59] recently discovered an important role of the bacterial genus *Variovorax* in attenuating the negative effects on root growth imposed by other bacterial isolates via modification of auxin concentration gradients in the rhizosphere. Plants also deploy extensive immune repertoires to ward off pathogens and control access of microbes to endophytic compartments [60], and some of our interactions may be related to ecotypic-specific resistance or susceptibility. Given the broad confidence intervals of our genome-wide scans, we resist the temptation to consider and discuss specific candidate genes, but imagine that identifying these molecular mechanisms will be an important direction in the field in coming years.

Approaches similar to ours are yielding positive insights across the field of plant–microbiome genetics. Several recent studies show the influence of microbiota on phenotypic plasticity, such as the regulation of flowering time in *Arabidopsis* [61] and morning glory [62]. O'Brien *et al*. [63] showed evidence that the effects of rhizosphere communities on trait variation likely played a role in the local adaptation of teosinte populations. In this study, we show that the interaction of ‘home’ and ‘away’ microbial communities differentially impacts the genetic architecture of *Panicum hallii* traits. This pattern sheds light on the role biotic factors may have played in the ecotypic divergence of *P. hallii*, and raises questions about the mechanisms through which microbes impact the genetic architecture of plant quantitative traits. Further work in this system may involve reductionist methods, including targeted inoculations of microbial strains; and reverse genetic approaches, which could both be used to identify specific mechanisms underlying our observed plant–microbe interactions.

## Data Availability

Raw sequencing reads are deposited at the NCBI Short Read Archive (https://www.ncbi.nlm.nih.gov/sra) under the BioProject PRJNA898795. Scripts are available online: https://github.com/AlbinaKh/QTL_P.hallii_Native_Microbiome. The data are provided in the electronic supplementary material [64].
